# Chiral Recognition Mechanism of 2,13-Bis(hydroxymethyl)-[7]thiaheterohelicene on Ag(111) Investigated by STM and MD Simulation

**DOI:** 10.3390/ijms262311458

**Published:** 2025-11-26

**Authors:** Changqing Ye, Takuma Hattori, Yuji Hamamoto, Pawel Krukowski, Akira Saito, Hideji Osuga, Yoshitada Morikawa, Yuji Kuwahara

**Affiliations:** 1 Department of Precision Engineering, Graduate School of Engineering, The University of Osaka, 2-1 Yamada-oka, Suita 565-0871, Osaka, Japan; yechangqing@ss.prec.eng.osaka-u.ac.jp (C.Y.); hattori@prec.eng.osaka-u.ac.jp (T.H.); hamamoto@c.oka-pu.ac.jp (Y.H.); saito@prec.eng.osaka-u.ac.jp (A.S.); morikawa@prec.eng.osaka-u.ac.jp (Y.M.); 2 Department of Communication Engineering, Okayama Prefectural University, 111 Kuboki, Soja 719-1197, Okayama, Japan; 3 Department of Solid State Physics, Faculty of Physics and Applied Informatics, University of Lodz, Pomorska 149/152, 90-236 Lodz, Poland; pawel.krukowski@uni.lodz.pl; 4 Faculty of Systems Engineering, Wakayama University, 930 Sakaedani, Wakayama 640-8510, Wakayama, Japan; osuga@sys.wakayama-u.ac.jp

**Keywords:** chiral recognition, molecular dynamics simulation, helicene, STM

## Abstract

We investigated the adsorption and aggregation properties of 2,13-bis(hydroxymethyl)-[7]thiaheterohelicene ([7]TH-diol) on the Ag(111) surface by scanning tunneling microscopy (STM) and molecular dynamics (MD) simulation. STM observation revealed that both racemic and enantiopure [7]TH-diol formed apparently similar “zigzag” chain structures. To elucidate the molecular arrangements in these structures, MD simulation successfully differentiates the formation mechanisms of these structures, demonstrating that hetero-chiral chains are stabilized primarily by van der Waals forces, whereas homo-chiral chains are stabilized through hydrogen bonding. The formation of homo-chiral chains is driven by the alignment of hydroxymethyl groups between the neighboring molecules, whereas the steric hindrance of helical skeletons affects chain growth. These findings highlight the critical role of inter-molecular interactions—particularly hydrogen bonding—in the self-assembly of helicene molecules.

## 1. Introduction

One of the most intriguing aspects of chirality is its role in molecular recognition processes, where enantiomers often interact selectively with their environment. Chiral recognition underpins many phenomena, from enantioselective catalysis [1,2] to the self-assembly of molecules [3,4,5], making it a critical area of research in surface and materials science.

Although chiral molecules are prevalent in nature, artificial chiral compounds offer unique opportunities to study various characteristics of chirality in controlled systems. Helicene, a class of ortho-condensed polycyclic aromatic compounds with a helical structure, is a typical example of chiral molecules. Owing to steric hindrance between tailing aromatic rings, helicene has a twisted, then screw-like structure, and eventually forms distinct alternating enantiomers. This inherent structural chirality of helicenes makes them an ideal platform for exploring the chiral recognition mechanisms in both solution- and surface-based systems [6,7,8,9,10].

One promising approach to understanding chiral recognition mechanisms is to study two-dimensional molecular layers formed on substrate surfaces, which facilitates a better understanding of how single molecules recognize each other [5,11,12,13,14,15]. Self-assembled structures of helicenes on surfaces have been extensively investigated by scanning tunneling microscopy (STM) [10,16,17,18,19,20,21,22], which provides exclusively high resolution for directly observing molecules in a real space. In this concept, thus far, many distinct molecular arrangements of helicenes by STM measurement have been reported. The adsorption of the racemic (*rac*-) [7]carbohelicene ([7]CH) on the (111) surfaces of Cu, Ag, and Au substrates resulted in similar zigzag chain structures with different lattice constants depending on the substrate used [17,22]. In contrast, (*M*)-type ((*M*)-) [7]CH formed homo-chiral trimers that fully covered the same substrates, reflecting the three fold symmetry of the surfaces [16,17]. On the Ag(100) surface, *rac*-[7]CH forms homo-chiral tetramers at low coverage and shows a transition from homo-chiral motifs to larger hetero-chiral zigzag chains at a nearly saturated coverage [18]. The diversity of these ordered structures arises from the interplay between inter-molecular and molecule–substrate interactions. In contrast, the substrate metal surfaces appear to have a relatively minor contribution to the ordered structure formation of racemic helicene; that is, the relevant chain structures do not show the affinity of the surface symmetry. This tendency is also observed in different [7]CH derivatives, whose racemic mixtures were found to form zigzag chain structures with alternating (*M*)-type and (*P*)-type enantiomers on the Ag(111) and Au(111) surfaces [23]. The adsorption of *rac*-5-amino[6]helicene at the interface between 1,2,4-trichlorobenzene and the Au(111) surface resulted in the formation of an intricate six fold symmetry network composed of hetero-chiral dimers [24], whereas enantiopure (*M*)- and (*P*)-5-amino[6]helicene self-assembles into homo-chiral trimers under the same conditions [25]. To the best of our knowledge, racemic and enantiopure helicenes exhibit different ordered structures on solid surfaces, where the properties of the substrate more likely affect the motifs formed by enantiopure helicenes.

Many studies have also focused on heterohelicenes, which consist of one or more different types of aromatic rings. In particular, thiaheterohelicenes, composed of alternant thiophene and benzene rings, have gained significant interest due to their easy functionalization at the terminal thiophene rings, which makes them ideal candidates for studying the effect of functional groups on molecular recognition. *Rac*-[7]thiaheterohelicene ([7]TH) was found to form hetero-chiral zigzag chains on the Ag(111) surface [19]. These chain structures are similar to those formed by [7]CH on the same surface. *Rac*-2,13-diformyl[7]thiaheterohelicene ([7]TH-CHO) showed well-ordered zigzag chains on the Ag(111) surface, which was similar to [7]TH [26]. Additionally, (*M*)-2,13-diphenyl[7]thiaheterohelicene showed hexagonal motifs on the Ag(111) surface, which was driven by strong molecule–substrate interactions [20]. On the other hand, (*M*)-2,13-bis(hydroxymethyl)-[7]thiaheterohelicene (dihydroxymethyl[7]thiaheterohelicene; [7]TH-diol) showed zigzag chain structures on the Au(111) surface. The molecular model proposed by Chaunchaiyakul et al. suggests that these structures arise without significant contributions from hydrogen bonding or strong π–π interactions between the molecules [21].

Although the behavior of helicenes on metal surfaces has been widely studied, the reason behind the differences in their ordered structures remains unclear. This is mainly because it is difficult to identify the chirality of each enantiomer and determine their molecular orientations. Theoretical calculations provide us great contributions to clarify the molecular arrangement on surfaces more precisely and evaluating the molecule–molecule and/or molecule–substrate interactions. The precise arrangements of helicene molecules on various surfaces have been determined by several researchers by density functional theory (DFT) calculation and molecular dynamics (MD) simulation [23,27,28,29,30]. The formation mechanism of chain structures of [7]TH on the Ag(111) surface was evaluated at the initial coverage by the MD simulation [30], revealing that the molecular arrangements were primarily driven by the π–π interaction between neighboring molecular skeletons.

**Figure 1:**
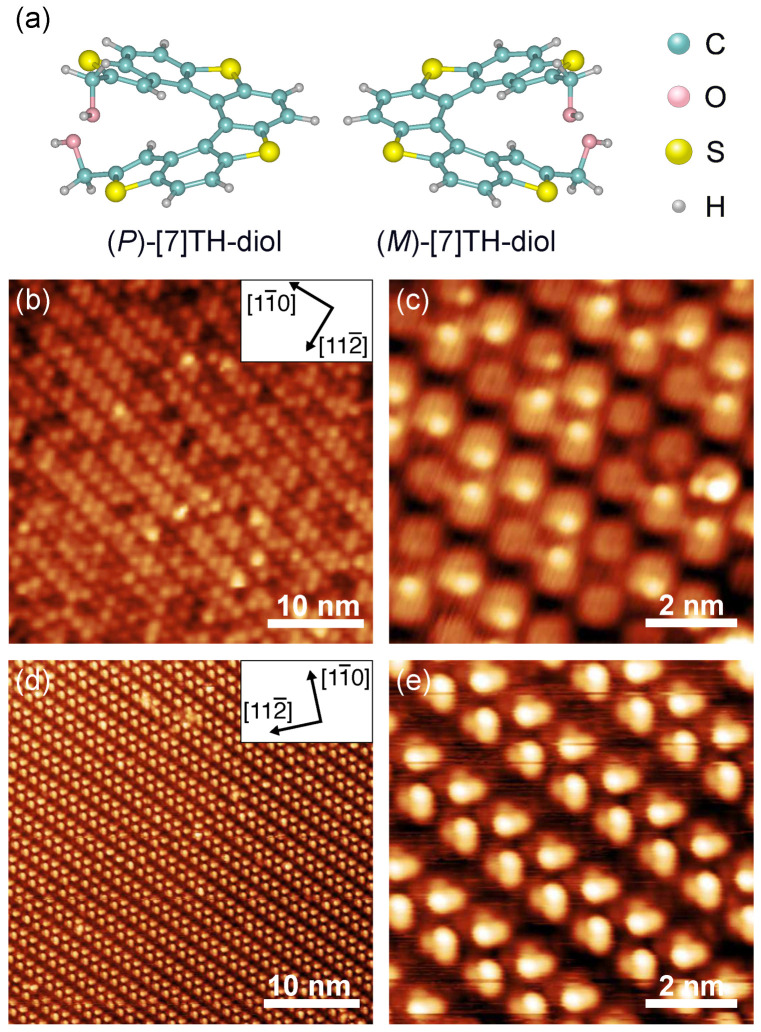
(**a**) Ball-and-stick models of (*M*)- and (*P*)-[7]TH-diols. The structures were optimized by DFT calculation. The cyan, pink, yellow, and white balls represent the C, O, S, and H atoms, respectively. (**b**,**c**) STM images of the self-assembled structures of *rac*-[7]TH-diol on the Ag(111) surface. (**d**,**e**) STM images of the self-assembled structures of (*M*)-[7]TH-diol on the Ag(111) surface. The STM images were obtained at (**b**) 1.0 V, 20 pA, (**c**) 1.0 V, 20 pA, (**d**) −2.5 V, 25 pA, and (**e**) −2.5 V, 25 pA.

In this work, we focus on the [7]TH-diol, whose molecular structures are illustrated in Figure 1a. An STM study of (*M*)-[7]TH-diol on the Au(111) surface highlighted a delicate interplay between molecule–molecule and molecule–substrate interactions [21], where the formation of homo-chiral chains reduced the influence of surface symmetry on molecular motifs. Here, we combine STM experiments and MD simulations to investigate the adsorption behavior of [7]TH-diol on the Ag(111) surface. The hydroxymethyl groups at the terminal of the molecular skeleton are expected to facilitate hydrogen bonding with its neighboring molecules and to modulate the molecular arrangement of ordered structures. To elucidate the role of hydroxymethyl groups in chiral recognition, we separately deposited *rac*- and (*M*)-[7]TH-diols on the Ag(111) surface. STM observation revealed the similar chain structures formed by both *rac*- and (*M*)-[7]TH-diols on the Ag(111) surface. To resolve the molecular arrangements within these structures, we also employed MD simulation to reproduce the observed structures and to evaluate the contributions of inter-molecular interactions and hydrogen bonding. Our results demonstrate that visually similar structures arise from different molecular arrangements, highlighting the critical role of hydroxymethyl groups in directing molecular recognition of chiral molecules and structure formation.

## 2. Results and Discussion

### 2.1. STM Observation of Self-Assembled Structures of [7]TH-diol on Ag(111)

After depositing *rac*-[7]TH-diol on the Ag(111) surface, the molecules self-assembled into well-ordered islands on the terraces of the Ag(111) surface, as shown in Figure 1b. The ordered islands were characterized as zigzag chains, with the chain directions tilted clockwise or anticlockwise (${20\pm 5)}^{\circ }$ from the <1$\bar{1}$0> direction of the substrate surface. The distance between the neighboring molecules along the chain direction was (1.2±0.1) nm. The density of molecules of the zigzag chain structures was (0.69±0.04) ${\mathrm{molecules}/\mathrm{nm}}^{2}$, which we defined as 1 monolayer (ML). These ordered molecular islands were observed only when the surface coverage of the molecules reached ∼1 ML. At lower coverages, molecular resolution was degraded due to the high mobility of the molecules on the surface (Figure S2). Similar chain structures were also observed for [7]TH and [7]TH-CHO on the Ag(111) surface [19,26]. The circular protrusions represent individual molecules in Figure 1c, in which small bright spots are randomly distributed inside these protrusions and were shifted close to the twin rows. These randomly distributed bright spots suggest the highest parts of individual molecules, possibly the hydroxymethyl groups away from the surface with different orientations.

On the other hand, (*M*)-[7]TH-diol was observed to form chain structures on the Ag(111) surface, as shown in Figure 1d. The chain directions were tilted clockwise or anticlockwise (${31\pm 10)}^{\circ }$ from the <1$\bar{1}$0> direction. Figure 1e shows an enlarged STM image of the chain structures, where each bright protrusion represents a single molecule. The distance between the neighboring molecules along the chain was (1.1±0.1) nm and the density of molecules was (0.74±0.06) ${\mathrm{molecule}/\mathrm{nm}}^{2}$. Further contrast within the protrusions reveals an off-centered bright spot in each molecule. The equally distributed bright spots suggest that the chain structures were composed of (*M*)-[7]TH-diol with the same orientation. Although the overall chain morphology closely resembled those formed by *rac*-[7]TH-diol, notable differences were observed. The chains formed by (*M*)-[7]TH-diol exhibited a larger tilting angle relative to the substrate, a shorter inter-molecular distance, and consequently a higher packing density, indicating a more compact molecular arrangement. These structural differences suggest that chain conformations of *rac*- and (*M*)-[7]TH-diols may be inherently distinct. Note that enantiopure carbohelicene molecules usually form trimers on the (111) surfaces or tetramers on the (100) surfaces of Cu, Ag, and Au substrates, with reflecting the surface symmetry [16,17,25]. This is in contrast to (*M*)-[7]TH-diol forming zigzag chains on the Ag(111) and Au(111) surfaces [21]. This suggests that molecular recognition among [7]TH-diols is largely affected by the hydroxymethyl groups, and the molecule–substrate interaction becomes less significant for (*M*)- or (*P*)-[7]TH-diol. The sulfur atoms within the helical skeleton have been expected to affect the adsorption behavior on the Ag(111) surface; however, our X-ray photoelectron spectroscopy (XPS) measurements and DFT calculations have confirmed that thiaheterohelicene is physisorbed on Ag(111), indicating weak molecule–substrate interactions [19,30]. Therefore, the sulfur atoms are not expected to dominate the adsorption geometry or adsorption properties, and the formation of the ordered structures observed by STM is mainly governed by inter-molecular interactions and the steric effects of the helical framework.

### 2.2. MD Simulation of [7]TH-diol

To elucidate the formation mechanisms of the self-assembled zigzag chains of [7]TH-diol on the Ag(111) surface, MD simulations were conducted for both racemic and enantiopure systems to investigate the molecule–molecule interaction in the ordered structures. Our XPS and DFT results have shown that [7]TH is certainly physisorbed on the Ag(111) surface [19,30]. As observed by STM, [7]TH and [7]TH-diol were highly mobile at low coverages on the Ag(111) surface, due to weak adsorption and the influence of the STM tip. These findings suggest that functionalization by hydroxymethyl groups at the terminals does not significantly affect the adsorption properties of [7]TH-diol on the Ag(111) surface.

Accordingly, inter-molecular interactions are therefore expected to play the dominant role in determining the arrangement of self-assembled structures. To better capture these intrinsic interactions while minimizing substrate effects, we employed a single-layer graphene sheet in the MD simulation, as graphene provides a chemically inert and structurally simple platform with minimal influence on molecular assembly. In addition, the GAFF force field used in this study is parameterized for organic and biomolecular systems and does not include reliable parameters for metallic atoms such as silver. As a result, directly modeling the Ag(111) surface would not provide reliable molecule–substrate interactions within this framework. Graphene, on the other hand, is widely adopted as a weakly interacting substrate in MD simulations, offering a well-defined and computationally compatible model that reproduces the essential physisorption characteristics observed experimentally on Ag(111). This approach allow us to isolate and analyze the intrinsic inter-molecular interactions governing the experimentally observed chain formation [31].

#### 2.2.1. Dimer Conformations

First, one (*M*)-type and one (*P*)-type enantiomers, as well as two (*M*)-type enantiomers, were randomly placed on the graphene substrate to elucidate the aggregation behavior of two molecules. When the system was kept at 275 K, the molecules migrated on the surface and finally formed dimers. It was found that [7]TH-diol was adsorbed with one terminal lying nearly parallel to the surface plane (Figure S3), in which the perpendicular distance between the center of the second benzene ring and the substrate was 3.5 Å, which is almost the same as that for [7]TH on graphene [30].

**Figure 2:**
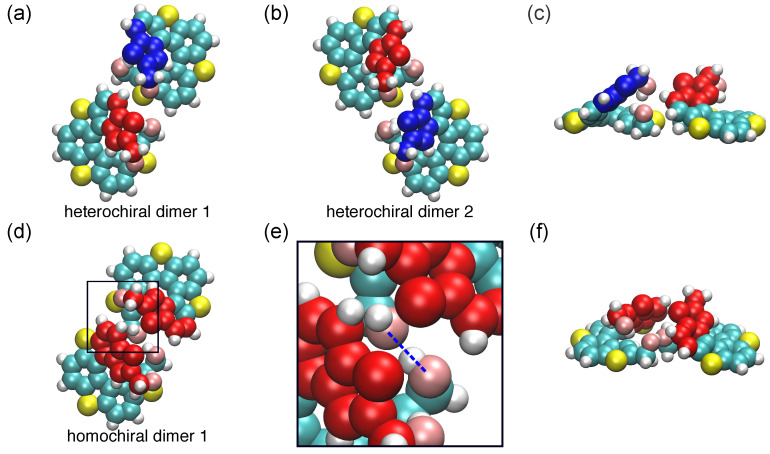
(**a**,**b**) Top views of the most favorable hetero-chiral dimers of [7]TH-diol. To distinguish enantiomers, the tops of (*M*)-type and (*P*)-type molecules are shown in red and blue, respectively. (**c**) Side view of hetero-chiral dimer 1. (**d**) Top view of the most favorable homo-chiral dimer of [7]TH-diol. (**e**) Enlarged image of the lower hydroxymethyl groups of two molecules, the dashed blue line represents the hydrogen bond forming between the molecules. (**f**) Side view of the homo-chiral dimer 1.

We traced the trajectories of two molecules throughout the simulation and evaluated their relative alignments at 80 K using 50 different initial configurations. In the case of a pair of (*M*)-type and (*P*)-type enantiomers, several different dimers were identified, and two of the most favorable configurations are shown in Figure 2a,b, which we refer to here as hetero-chiral dimers 1 and 2. These dimers had similar adsorption energy $E_{\mathrm{ads}}$ values of (−1.327±0.010) eV per molecule, with formation probabilities of 38% and 32% for hetero-chiral dimers 1 and 2, respectively. Notably, hetero-chiral dimers 1 and 2 show mirror images. The slight difference between these probabilities could be attributed to statistical deviations. Hetero-chiral dimers are characterized by the “face-to-face” configuration, where the enantiomers rotate by approximately $180^{\circ }$ relative to each other. This arrangement prevents the partial overlap between the upper parts of one molecule and the lower parts of the other owing to the presence of hydroxymethyl groups at the terminals. The –OH groups are oriented inward toward the helical structures of the molecules, and the O…H distance between the nearest –OH groups of the two molecules is longer than 4.00 Å. The side view of hetero-chiral dimer 2 in Figure 2c indicates that the interaction between the spiral skeletons, primarily van der Waals interaction, plays a significant role in the formation of hetero-chiral dimers of [7]TH-diol. The other hetero-chiral dimer configurations are shown in Figure S3. Two enantiomers in these dimers aligned towards the same direction, which was similar to the case of [7]TH dimers [30]. However, the steric hindrance between hydroxymethyl groups lead two enantiomers having a longer inter-molecular distance; and therefore, these dimers are less stable than hetero-chiral dimer 1 or 2 in interaction energy.

In the case of two (*M*)-type enantiomers, only one unique homo-chiral dimer was identified as a stable structure (Figure 2d). The $E_{\mathrm{ads}}$ of the dimer was calculated to be (−1.371±0.010) eV per molecule, indicating that the homo-chiral dimer was slightly more stable than the hetero-chiral ones. In the homo-chiral dimer, the (*M*)-type molecules rotate approximately $90^{\circ }$ relative to each other, with the upper parts of their helical skeletons standing upright and facing one another (Figure 2f). Additionally, it was found that the –OH groups proximal to the surface had a specific orientation, with an angle about $162^{\circ }$ between O…H–O and a O…H distance of 1.86 Å between the nearest –OH groups, which strongly indicates hydrogen bond formation between the two enantiomers (Figure 2e) [32]. Importantly, while the lower –OH groups participate in hydrogen bonding within the dimer, the upper –OH groups remain exposed and face outward. This dimer configuration allows additional hydrogen bonding interactions when another enantiomer approaches the dimer. When a third molecule comes into proximity, its upper –OH groups can interact with outward-facing –OH groups of the dimer. This interaction extends the hydrogen bond network among enantiopure [7]TH-diol, facilitating the formation of larger assemblies and potentially affecting the growth direction of the chain structures.

#### 2.2.2. Formation of Chain-like Structures

Second, we increased the number of molecules (*n*) in the MD simulation to clarify the formation mechanism of the zigzag chain structures observed in both the hetero-chiral and homo-chiral systems. As *n* increased, the molecules tended to aggregate into clusters. When *n* reached 4, the molecules formed tetramers with various molecular arrangements, in which the majority of hetero-chiral and homo-chiral tetramers are shown in Figure S4. Note that hydroxymethyl groups always come close to each other to form hydrogen bonds with their neighbors, and there was at least one hydrogen bond in the tetramers. Although the tetramers maintained hydrogen bonding between hydroxymethyl groups, their molecular arrangements did not directly extend the dimer configurations in a one-dimensional manner. This structural deviation was likely due to changes in the inter-molecular interaction and increased steric constraints as additional molecules approached and assembled.

**Figure 3:**
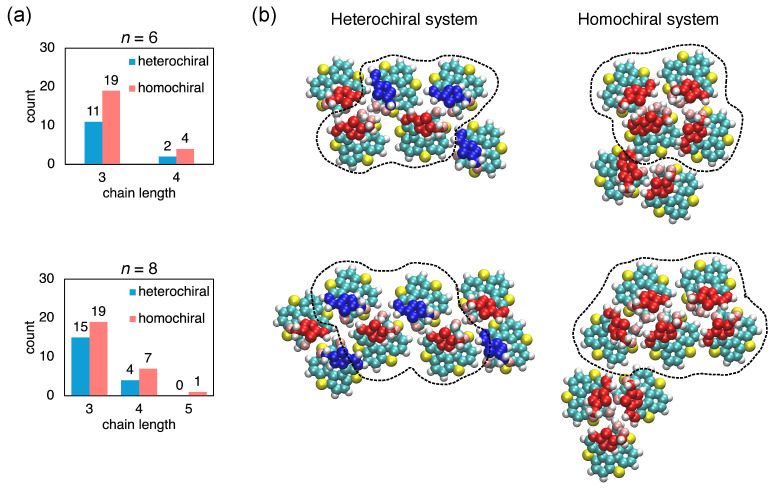
(**a**) Histograms of chain-like structures found for systems composed of n=6 molecules (upper), and n=8 molecules (bottom). The chain length is defined by the number of molecules arranged in the chain-like structure. (**b**) Molecular arrangements containing chain-like structures found in hetero-chiral (upper panel) and homo-chiral (bottom panel) systems. The systems are composed of n=6 or 8 molecules. The frames with dashed lines represent the chain-like structures.

To gain more insight into these chain-like arrangements, we conducted a population analysis based on 50 independent simulations with different initial molecular configurations, with the results presented as histograms in Figure 3a for larger clusters with n=6 or 8. Here, we define chain length as the number of molecules arranged in dimer configurations. Some molecules were arranged into chain-like structures, as shown in Figure 3b. The nearest-neighbor arrangements in these chains closely resembled those observed in hetero-chiral and homo-chiral dimers, suggesting that dimer interactions play a key role in the formation of extended structures. We predominantly observed very short chain arrangements existing in the molecular clusters composed of three or four molecules arranged in chain-like structures. Owing to the presence of surrounding molecules, the molecules in short chains slightly adjust their relative orientations. The molecules were initially placed at random positions on the substrate, which allowed for a high degree of freedom. Therefore, it was difficult for all molecules in the system to form a single, extended linear chain. Notably, the probability of forming chain structures in hetero-chiral systems was lower than that in homo-chiral systems. This phenomenon is attributed to a slight difference in $E_{\mathrm{ads}}$ between the hetero-chiral and homo-chiral dimer configurations. Additionally, in the hetero-chiral systems, the combination of one (*M*)-type and (*P*)-type molecules can lead to several different hetero-chiral dimers, further impeding the formation of extended chain-like structures. As a result, the molecules tend to become trapped in specific metastable configurations that are stable throughout the simulation, making it difficult for them to overcome the associated energy barrier to reorganize and facilitate chain elongation.

#### 2.2.3. Extended Molecular Chain Models

**Figure 4:**
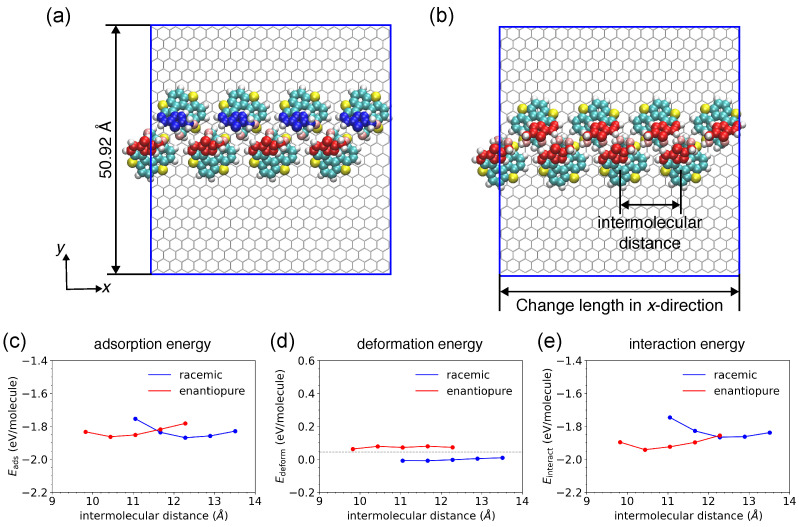
Molecular models of (**a**) racemic and (**b**) enantiopure chains on graphene, the inter-molecular distance is 13.50 Å. The chains align along the *x*-direction. Blue frames represent the unit cells used in the MD simulations. Energy profiles of (**c**) adsorption energy ($E_{\mathrm{ads}}$), (**d**) the molecular deformation energy of the molecules ($E_{\mathrm{deform}}$), (**e**) interaction energy ($E_{\mathrm{interact}}$) are plotted as functions of inter-molecular distance. The blue and red curves correspond to racemic and enantiopure chains, respectively. The dashed line in (**d**) represents the $E_{\mathrm{deform}}$ of the adsorption of a single [7]TH-diol on the graphene surface.

To grasp the inter-molecular interaction through the formation of long-range zigzag chains of [7]TH-diol, we intentionally prepared chain-like structures of eight molecules and used them to construct models under periodic boundary conditions. Initially, the molecules were set to be uniformly arranged along the chain that extended in the *x*-direction. In the hetero-chiral system (Figure 4a), each neighboring pair of (*M*)-type and (*P*)-type enantiomers has either the configuration of hetero-chiral dimer 1 or 2, with an alternating arrangement of different enantiomers along the racemic chain. In contrast, in the homo-chiral system (Figure 4b), the hydroxymethyl groups of (*M*)-type molecules gather toward the center of the enantiopure chain, bringing the –OH groups close together. This arrangement allows the lower and upper –OH groups to form alternating hydrogen bonds with neighboring molecules along the chain.

To obtain the optimized structure of the molecular chains, we systematically changed the distance between neighboring molecules by adjusting the unit cell length in the *x*-direction for the integer of multiples of the graphene lattice constant. For each configurations with different periodicities, the chain models were equilibrated at 1 K for 500 ps. The chain structures after equilibration are present in Figure S5. In Figure 4c, $E_{\mathrm{ads}}$ is plotted as a function of inter-molecular distance. It was found that the racemic chain exhibited its lowest $E_{\mathrm{ads}}$ of −1.869 eV with an optimized inter-molecular distance of 12.28 Å, whereas the enantiopure chain showed a slightly higher $E_{\mathrm{ads}}$ of −1.863 eV at 10.44 Å. Although the energy difference between the two chain configurations appears small on a per-molecule basis, it becomes more appreciable when considering extended chains, leading to a more distinct thermodynamic preference for the racemic arrangement. This small difference is comparable to the intrinsic uncertainty of the force field; however, the consistent trend observed across multiple chain lengths and its agreement with the experimentally observed geometry indicate that the calculated preference is physically meaningful. The small energy difference between the two configurations might also be important for chain formation at this low temperature. These inter-molecular distances are in good agreement with the STM observations, where the racemic and enantiopure chains exhibited distances along the chains of approximately (1.2±0.1) nm and (1.1±0.1) nm, respectively. However, since the simulation was performed on a graphene substrate with weak molecule–substrate interactions, the adsorbed [7]TH-diol had no significant preferred adsorption site. As a result, the specific chain orientation observed in STM images could not be fully reproduced. The optimized racemic and enantiopure chain models were then equilibrated at 80 K for 500 ps, and both structures remained stable (see Supplementary Materials, Video S1.)

In MD simulations, the total potential energy consists of two main components: bonded energy, which accounts for internal interactions within a molecule (such as bond stretching, angle bending, and dihedral torsions), and nonbonded energy, which includes interactions between atoms that are not directly bonded, such as van der Waals forces and electrostatic (Coulomb) interactions. The bonded energy reflects how the internal molecular structure contributes to the overall energy, whereas the nonbonded energy governs how molecules interact with each other and with the substrate.

Accordingly, the difference in bonded energy between adsorbed molecule and free molecules can be interpreted as the deformation energy ($E_{\mathrm{deform}}$, see Figure 4d). This deformation energy is calculated as the bonded energy of the adsorbed molecule subtract that of the free molecule, reflecting the structural distortion induced by adsorption and/or interactions with neighboring molecules. Similarly, the difference in nonbonded energy can be interpreted as the interaction energy ($E_{\mathrm{interact}}$, see Figure 4e), which includes contributions from both molecule–molecule and molecule–substrate interactions.

The total adsorption energy per molecule can therefore be expressed as

(1) $$E_{\mathrm{ads}}=E_{\mathrm{deform}}+E_{\mathrm{interact}}$$

As the average inter-molecular distance increased shown in Figure 4, corresponding to an increase in unit cell length, structural rearrangements were observed in both racemic and enantiopure chains to maintain favorable inter-molecular interactions. In racemic chains, (*M*)-type and (*P*)-type enantiomers approached each other along the *y*-direction, resulting in a new molecular arrangement to preserved inter-molecular distances (see Supplementary Materials, Video S2). In contrast, in enantiopure chains, an increment of the unit cell length led to a deviation from a uniform molecular distribution, in which molecules tended to form closely associated pairs (see Supplementary Materials, Video S3). Consequently, the average distance between the nearest-neighbor molecules remained nearly unchanged, and thus, $E_{\mathrm{deform}}$ remained nearly constant in both systems. These observations suggest that the inter-molecular interactions are primarily governed by nearest-neighbor interactions.

Notably, the absolute values of $E_{\mathrm{deform}}$ differ between the two systems: molecules in the enantiopure chain exhibit greater structural deformation than those in the racemic chain. This indicates that differences in molecular arrangement give rise to distinct inter-molecular interactions, which in turn induce molecular deformation to stabilize the overall structure. When a single molecule is adsorbed, it may undergo conformational changes, such as changes in bond angles, dihedrals, or internal strain, to optimize its interaction with the substrate. The $E_{\mathrm{deform}}$ for a single adsorbed molecule is shown as the dashed line in Figure 4d. Upon the adsorption of multiple molecules, additional inter-molecular interactions can further affect the molecular conformation. In the case of enantiopure chains, the larger $E_{\mathrm{deform}}$ than that of a single adsorbed molecule is mainly due to hydrogen bonding between the neighboring molecules, which might cause additional structural distortion for the adsorbates. In contrast, for racemic chains, $E_{\mathrm{deform}}$ is smaller than that of a single adsorbed molecule, suggesting that molecular deformation is reduced when multiple molecules are adsorbed together. The inter-molecular interactions in racemic chains appear to stabilize the adsorbed molecules in a way that decreases their distortions. This implies that neighboring molecules can support each other structurally, effectively reducing the strain on individual molecules and promoting a more energetically favorable structure.

The profile of $E_{\mathrm{interact}}$ (Figure 4e) shows that molecules in the enantiopure chain exhibit stronger inter-molecular interactions than those in the racemic chain. Since the conformational changes induced by the molecular adsorption on the surface should be identical for both (*M*)-type and (*P*)-type enantiomers, the molecule–substrate interactions must also be identical for both enantiomers. Therefore, the greater deformation and interaction energies observed in the enantiopure chain should originate from interactions between neighboring molecules rather than from interactions between the molecules and the substrate.

**Figure 5:**
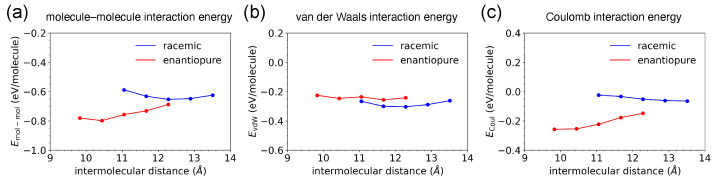
(**a**) Molecule–molecule interaction energy, (**b**) van der Waals interaction energy, and (**c**) Coulomb interaction energy are plotted as functions of inter-molecular distance. The blue and red curves represent racemic and enantiopure chains, respectively.

To further elucidate the role of inter-molecular interactions, we recalculated the potential energy of the adsorbates using the same molecular trajectories obtained from the MD simulations with excluding molecule–substrate interactions. This enabled us to determine the inter-molecular interaction energy, confirming that differences in molecular arrangements between racemic and enantiopure chains were primarily governed by inter-molecular forces. Figure 5a shows molecule–molecule interactions ($E_{\mathrm{mol}-\mathrm{mol}}$) of individual molecules within the chains.

To better understand how inter-molecular interactions affect molecular arrangements, $E_{\mathrm{mol}-\mathrm{mol}}$ was further divided into two components, namely van der Waals and Coulomb interactions, as shown in Figure 5b and Figure 5c, respectively. In the hetero-chiral system, the van der Waals interaction appeared domination in the formation of the racemic chain, because the opposite chirality of spiral structures of [7]TH-diol allowed the molecules to fit together effectively. In the homo-chiral system, the van der Waals interaction also played a crucial role in the formation of the enantiopure chain. Additionally, the Coulomb interaction significantly contributed to structural stabilization, which results from the hydrogen bonds forming between neighboring molecules. Besides the linear chain structures investigated in this work, inter-molecular interactions among chiral molecules can also give rise to more complex supramolecular architectures, including cyclic chiral assemblies [33].

#### 2.2.4. Effect of Different Partial Charge Distributions

Since hydrogen bonds between neighboring molecules were found to significantly affect the ordered structures of [7]TH-diol, especially in the case of zigzag chains formed by (*M*)-[7]TH-diol, we investigated how variations in partial atomic charges affect inter-molecular interactions in MD simulations. AM1-BCC charges were used as the reference, and the Mulliken charges obtained at the B3LYP/6-311++G(d,p) level with Gaussian09 were tested for comparison (see Supplementary Materials, Figure S6). The chains obtained using Mulliken charges with reduced –OH polarity failed to maintain ordered structures, indicating that partial charges significantly affect the formation and stability of molecular assemblies.

### 2.3. Role of Functional Groups in Molecular Arrangements

The periodicities along single zigzag chains from the experimental results were well reproduced by MD simulation in both the hetero-chiral and homo-chiral systems, which suggests that the inter-molecular interaction among [7]TH-diol was well captured by the models in the MD simulation. Chain-like structures formed from random initial configurations in the MD simulations on graphene do not show a peculiar orientation, while the experimentally observed chains on Ag(111) tend to align along specific directions relative to the step edges. This behavior highlights the role of the substrate in directing large-scale organization, indicating the limitation of using graphene as a substrate for the MD simulation. Despite this, the graphene-based simulations remain valuable for elucidating the inter-molecular recognition mechanisms that drive chain formation.

In the STM images, [7]TH-diol were observed as single circular protrusions, and there were extra off-centered bright spots inside the protrusions. These bright spots were inferred to as the upper parts of the molecules. Therefore, the STM images in Figure 1 can be interpreted as follows: In the hetero-chiral system, the neighboring molecules within the zigzag chains are primarily stabilized by the van der Waals interaction between their steric skeletons. In this case, the hydroxymethyl groups appear to retain a relatively high degree of rotational freedom during STM measurements. Because [7]TH-diol adsorbs flat on the surface, and only local rotations around the σ bonds of the hydroxymethyl groups occur. Such localized rotations modulate the apparent height of terminal groups without altering the overall footprint. The randomly distributed bright spots within the circular protrusions therefore indicate that the rotation of the hydroxymethyl groups is only partially restricted and that their orientation does not significantly affect the overall self-assembled structures. In contrast, in the homo-chiral system, hydrogen bonds form between the –OH groups of neighboring molecules, which restrict the rotation of the σ bonds connecting the hydroxymethyl groups to the helicene skeletons. As a result, the orientations of the hydroxymethyl groups become more fixed, leading to the uniform distribution of bright spots observed in the STM images.

Unlike other enantiopure helicenes on the (111) surface [16,17,25], which form trimer or tetramer motifs, (*M*)-[7]TH-diol assembled into zigzag chains, indicating that its molecular arrangement was primarily governed by inter-molecular interactions rather than substrate effects. Since the molecule–substrate interactions for helicene and its derivatives are expected to be similar, the key factor differentiating the observed structures is the nature of the inter-molecular interactions. In the above cases, the formation of small three-fold and four-fold motifs suggests that inter-molecular forces alone are insufficient to drive extended chain formation, and surface symmetry plays a role in guiding molecular arrangement. In contrast, for [7]TH-diol, the presence of hydroxymethyl groups likely introduced additional hydrogen bonding interactions, which strengthened inter-molecular attraction and promoted the formation of continuous chains, overriding any potential effect of surface symmetry. This suggests that functional groups play a crucial role in directing molecular assembly, even in cases where molecule–substrate interactions remain constant. This tendency is consistent with previous reports on physisorbed molecular assemblies, where the dominance of inter-molecular interactions over substrate coupling drives reorientation and long-range order in self-assembled domains [18,34,35].

We conducted STM observations using various thiaheterohelicene derivatives [19,20,21,26]. in which *rac*-[7]TH, *rac*-[7]TH-diol, and (*M*)-[7]TH-diol all form similar zigzag chains on the Ag(111) surface. Interestingly, our simulation results revealed that the molecular arrangement/conformation within the chain structures differ entirely in all three cases. MD simulations for [7]TH on graphene demonstrated that *rac*-[7]TH forms zigzag chains with alternating (*M*)-type and (*P*)-type enantiomers arranged “back on back” via the π–π interaction between neighboring molecules. In contrast, the steric hindrance induced by the hydroxymethyl groups at the terminals of [7]TH-diol prevents the similar conformation from [7]TH. As a result, *rac*-[7]TH-diol forms a “face-to-face” arrangement of alternating (*M*)-type and (*P*)-type enantiomers, with the chain stabilized by the van der Waals interaction between the spiral skeletons of neighboring molecules. The opposite conformation of neighboring molecules in the zigzag chains of *rac*-[7]TH-diol also leads to an inter-molecular distance along the chain larger than (11.1±0.1) Å observed for [7]TH by STM.

In the case of (*M*)-[7]TH-diol, STM observations show the formation of similar zigzag chains on the Ag(111) surface. The –OH groups of the molecules aggregate in the middle of chain structures, while, pointing outward of the molecules, enabling the formation of hydrogen bonds with adjacent molecules through the upper and lower –OH groups. As a result, the upper and lower hydrogen bonds alternate along the chains. Unlike the triangular or hexagonal structures typically formed by other helicene derivatives on the (111) surfaces of noble metal substrates [16,17,20], these chain motifs disregard the threefold symmetry of the (111) surfaces, suggesting that inter-molecular interactions dominate the formation of the ordered chain structures. The chain structures were also reproduced in the MD simulations performed on a graphene substrate, which exhibited weaker molecule–substrate interactions and nonspecific molecular orientations. These simulation results further emphasize the crucial role of inter-molecular interactions—specifically hydrogen bonds—in determining the structural arrangements.

## 3. Materials and Methods

All the experiments were carried out using a low-temperature ultrahigh-vacuum STM system (UNISOKU Co., Ltd., Hirakata, Osaka, Japan, USM1400) at a base pressure of less than $1.0\times 10^{-10}$ Torr. The scanning system was controlled by a Nanonis BP 5 controller (SPECS GmbH, Berlin, Germany). STM images were obtained at 80 K in constant-current mode using electropolished PtIr tips (UNISOKU Co., Ltd., Hirakata, Osaka, Japan) and analyzed using the Gwyddion [36] package. The Ag(111) films epitaxially grown on mica (Georg Albert PVD) were used as substrates. The Ag(111) surfaces were cleaned by repeated cycles of Ar_+_ ion sputtering and subsequent annealing at 633 K. The *rac*- and (*M*)-[7]TH-diols were thermally deposited separately at a pressure less than $3.0\times 10^{-8}$ Torr using a Knudsen-type organic molecular evaporator (KITANO SEIKI Co., Ltd., Ota, Tokyo, Japan) inside a preparation chamber. The *rac*- and (*M*)-[7]TH-diols were deposited on the substrates at 483 K and 510 K, respectively, while the substrates were held at room temperature. The synthesis and optical resolution of [7]TH-diol were described elsewhere [37]. Previous studies have shown that [7]TH possesses a much high activation barrier for racemization, and that the introduction of functional groups to the helical backbone does not significantly alter this activation energy [38]. Such a large barrier ensures that the helical configuration remains stable under our experimental conditions. Accordingly, we expect a similar activation barrier for [7]TH-diol, and its chirality should be well preserved during the sublimation process in the experiments.

While DFT calculations are highly accurate for describing small systems and local adsorption structures [39], they become computationally demanding for large-scale molecular assemblies. For example, modeling two [7]TH-diol molecules based on STM dimensions requires more than twice the number of Ag atoms compared to a single-molecule model, with computational cost increasing roughly with the cube of the number of electrons. In such cases, MD or Monte Carlo simulations provide an efficient approach to capture collective molecular behavior and thermodynamic stability [40]. Therefore, in this study, MD simulations were employed to investigate the inter-molecular interactions that drive the formation of self-assembled chains.

MD simulation was performed with the LAMMPS [41] program. A flat Ag(111) surface was approximated by a monolayer graphene sheet with a lattice constant of 2.46 Å. To investigate the aggregation behavior of [7]TH-diol on graphene at a low surface coverage, the size of a unit cell (*xy*-plane) was changed with a constant molecular number density of approximately 0.2 ${\mathrm{molecules}/\mathrm{nm}}^{2}$, which corresponded to a different number of molecules in the system (see Figure S1). To investigate the formation of long-range zigzag chains, the *x*-dimension was changed to be commensurate with the periods of chain structures, and the y-dimension was fixed at 51.05 Å. The perpendicular dimension (*z*) was 40.00 Å and kept orthogonal to the *xy*-plane in all the cases. Periodic boundary conditions were applied in all three directions. Initially, the molecules were randomly positioned on the graphene substrate, and the distances between the geometric center of molecules and the substrate were set at 5.00 Å.

The general AMBER force field (GAFF) [42,43] was employed to describe the interaction between atoms. The force fields used in this study all rely on fixed partial charges to describe Coulomb interactions and on the Lennard–Jones 6–12 model to describe van der Waals interactions. The Coulomb interaction energy $E_{\mathrm{Coul}}$ and van der Waals interaction energy $E_{\mathrm{vdW}}$ are presented as,

(2) $$E_{\mathrm{Coul}}=\sum_{i=1}^{N}\sum_{j>i}^{N}\frac{q_{i}q_{j}}{4\pi \varepsilon_{0}r_{ij}}$$

(3) $$E_{\mathrm{vdW}}=\sum_{i=1}^{N}\sum_{j>i}^{N}4\epsilon_{ij}\left[{\left(\frac{\sigma_{ij}}{r_{ij}}\right)}^{12}-{\left(\frac{\sigma_{ij}}{r_{ij}}\right)}^{6}\right]$$

$r_{ij}$ represents distance between two atoms *i* and *j*. Partial charges $q_{i}$ and $q_{j}$ were calculated using the AM1-BCC algorithm [44,45] and are listed in Table S1. $\epsilon_{0}$ is vacuum permittivity. *N* is total number of atoms. The Lennard–Jones interaction parameters $\sigma_{ii}$ and $\epsilon_{ii}$ were obtained from AMBER force field [42,43]. For unlike atom pairs, the Lennard–Jones parameters were calculated using the Lorentz–Berthelot rules. Note that partial charges and Lennard–Jones parameters were not assigned to the carbon atoms in the graphene sheet. The graphene substrate was frozen, and the structures of adsorbates were fully relaxed while the SHAKE algorithm [46] was adopted to keep C–H bonds rigid during the simulation. The molecules’ temperature was controlled using the Nosé–Hoover thermostat [47,48] with a time step of 1 fs. After the initial energy minimization, the system was equilibrated at 275 K for 2 ns and cooled to 80 K at a controlled cooling rate of 100 K/ns. After cooling, the system was equilibrated at 80 K for 0.5 ns, and thermal dynamic data were sampled every 0.1 ps during this period for further analysis.

The adsorption energy of molecular clusters on the surface was defined as

(4) $$E_{\mathrm{ads}}=\left(E_{\mathrm{molecule}/\mathrm{substrate}}-nE_{\mathrm{molecule}}-E_{\mathrm{substrate}}\right)/n$$

where $E_{\mathrm{molecule}/\mathrm{substrate}}$, $E_{\mathrm{molecule}}$, and $E_{\mathrm{substrate}}$ represent the potential energies of the adsorption system, isolated free molecule, and isolated substrate, respectively; *n* is the number of molecules included in the adsorption system.

## 4. Conclusions

In this study, we investigated the self-assembled structures of *rac*- and (*M*)-[7]TH-diol on the Ag(111) surface by both STM observation and MD simulation. Unlike other helicenes or their derivatives, both *rac*- and (*M*)-[7]TH-diols formed identical zigzag chains. Notably, the chain structures formed by the (*M*)-[7]TH-diol on the Ag(111) surface were not affected by the surface symmetry, in contrast to other enantiopure helicene derivatives, which typically form triangular or hexagonal motifs reflecting the threefold surface symmetry. Our findings demonstrate that although [7]TH-diol maintains adsorption characteristics similarly to [7]TH owing to weak molecule–substrate interactions, the hydroxymethyl groups significantly affect inter-molecular interactions, particularly in homo-chiral systems. The hydroxymethyl groups facilitate hydrogen bonding, which stabilizes homo-chiral dimers and enables their assembly into chain-like structures. In contrast, the van der Waals interaction between spiral skeletons dominates in hetero-chiral systems, where steric hindrance prevents hydrogen bond formation. The zigzag chain motifs observed in STM images were reproduced in MD simulations, confirming that inter-molecular interactions play a dominant role over molecule–substrate interactions. Notably, the structural differences between [7]TH and [7]TH-diol chains highlight the significant effect of terminal functional groups. These insights enhance our understanding of the relationship between the molecular structure and the self-assembly, providing a foundation for designing surface-adsorbed chiral systems with tailored properties.

## Data Availability

The original contributions presented in this study are included in the article/Supplementary Materials. Further inquiries can be directed to the corresponding author(s).

## Supplementary Materials

The supporting information can be downloaded at: https://www.mdpi.com/article/10.3390/ijms262311458/s1. References [44,45,49] are cited in the Supplementary Materials.

## Abbreviations

The following abbreviations are used in this manuscript:

*rac*	racemic
[7]TH	[7]thiaheterohelicene
[7]TH-diol	2,13-bis(hydroxymethyl)-[7]thiaheterohelicene
[7]CH	[7]carbohelicene
STM	scanning tunneling microscopy
ML	monolayer
MD	molecular dynamics
DFT	density functional theory
XPS	X-ray photoelectron spectroscopy
