# Supplementary material for: Chiral Recognition Mechanism of 2,13-Bis(hydroxymethyl)-[7]thiaheterohelicene on Ag(111) Investigated by STM and MD Simulation
Source: Int J Mol Sci. 2025 Nov 26;26(23):11458. doi: 10.3390/ijms262311458 (PMC12692249; doi:10.3390/ijms262311458)
Supplement: Supplementary file 1 [file ijms-26-11458-s001.zip › ijms-3949154-supplementary/ESI/supporting.pdf]

Electronic Supplementary Information

Chiral Recognition Mechanism of 2,13-Bis(hydroxymethyl)[7]thiaheterohelicene on Ag(111) investigated by STM and MD simulation

Changqing Ye,[1] Takuma Hattori,[1] Yuji Hamamoto, [1,2] Pawel Krukowski, [3] Akira Saito,[1] Hideji Osuga,[4] Yoshitada Morikawa,[1] and Yuji Kuwahara[1,*]

[1] Department of Precision Engineering, Graduate School of Engineering, The University of Osaka, 2-1 Yamada-oka, Suita 565-0871, Osaka, Japan; yechangqing@ss.prec.eng.osaka-u.ac.jp (C.Y.); hattori@prec.eng.osaka-u.ac.jp (T.H.); hamamoto@c.oka-pu.ac.jp (Y.H.); saito@prec.eng.osaka-u.ac.jp (A.S.); morikawa@prec.eng.osaka-u.ac.jp (Y.M.); kuwahara@prec.eng.osaka-u.ac.jp (Y.K.)

[2] Department of Communication Engineering, Okayama Prefectural University, 111 Kuboki, Soja 719-1197, Okayama, Japan

[3] Department of Solid State Physics, Faculty of Physics and Applied Informatics, University of Lodz, Pomorska 149/152, 90-236 Lodz, Poland; pawel.krukowski@uni.lodz.pl

[4] Faculty of Systems Engineering, Wakayama University, 930 Sakaedani, Wakayama 640-8510, Wakayama, Japan; osuga@sys.wakayama-u.ac.jp

[*] Correspondence: kuwahara@prec.eng.osaka-u.ac.jp

Table of Contents

1. Unit cells used in MD simulation.
2. STM image of *rac*-[7]TH-diol on Ag(111) at low coverage.
3. Heterochiral dimer configurations.
4. Typical heterochiral and homochiral tetramers.
5. Chain structures after equilibration at 1 K.
6. Partial atomic charges of [7]TH-diol.

1. Unit cells used in MD simulation.

The aggregation behavior of [7]TH-diol molecules on graphene was investigated under low surface coverage. To maintain a constant molecular number density ($\approx$ 0.2 molecules/nm^2), the unit cell size in the *xy*-plane was adjusted in response to the number of molecules in the system. Initially, the molecules were randomly placed on the surface.

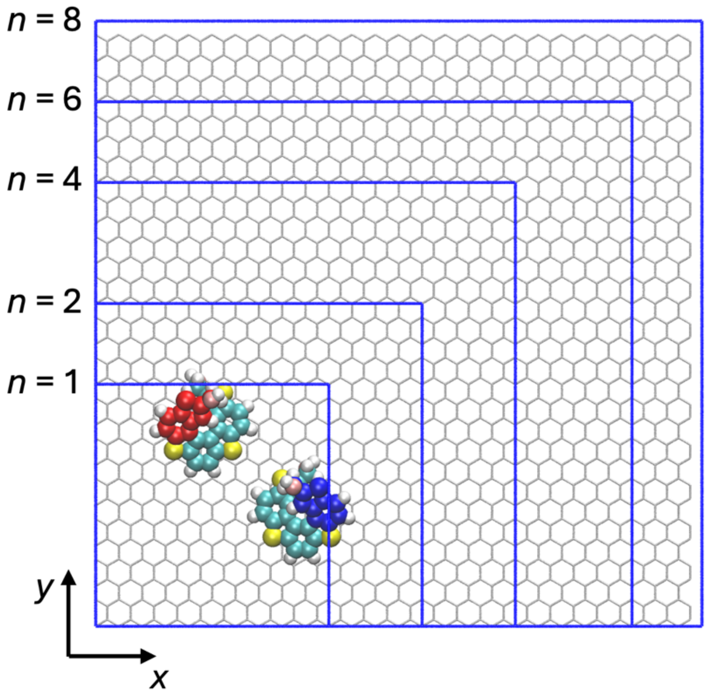

Figure S1. Unit cells used in MD simulations when the number of molecules (n) is different: 24.56 × 25.52 Å, 34.38 × 34.03 Å^2, 39.30 × 38.29 Å^2, 44.21 × 46.79 Å^2, 56.49 × 55.30 Å^2, 63.86 × 63.81 Å^2 with the number of molecules n = 1, 2, 4, 6, 8, respectively. The initial configuration of molecules is shown for n = 2 case.

2. STM image of *rac*-[7]TH-diol on Ag(111) at low coverage.

At low coverages, only disordered areas were observed, and the molecules showed high mobility on the terrace of the Ag(111) surface.

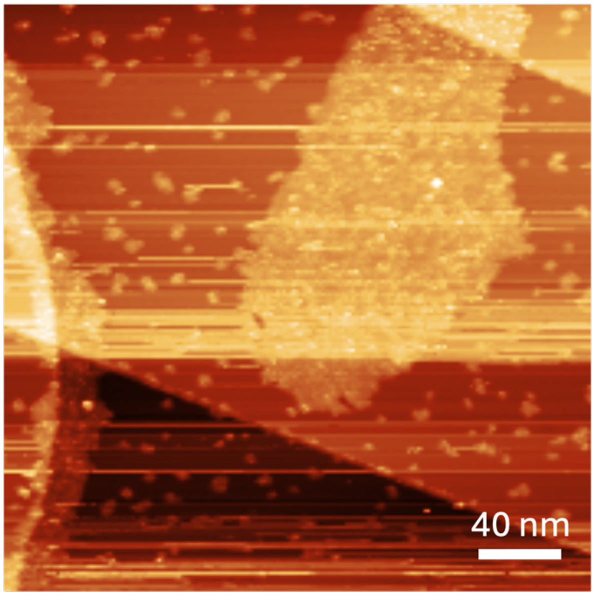

Figure S2. STM image of *rac*-[7]TH-diol on Ag(111) at 1 V, 20 pA. The molecules move on the terraces of the surface, appearing as streaks due to the dragging the molecules by the tip scanning in the STM image.

3. Heterochiral dimer configurations.

Two different enantiomers form multiple heterochiral dimer configurations. Shown here are two additional heterochiral dimers (dimers 3 and 4) with lower formation probabilities. These configurations are also mirror images of each other. Unlike dimers 1 and 2, the enantiomers in dimers 3 and 4 are aligned in the same direction.

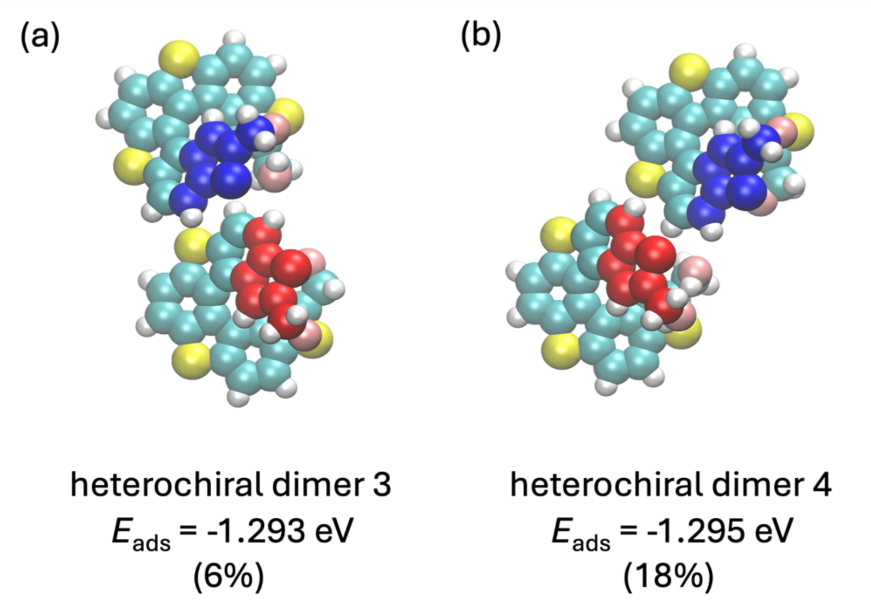

Figure S3. Heterochiral dimers found in MD simulation.

4. Typical heterochiral and homochiral tetramers.

When the number of molecules increases to four, they assemble into tetramers showing diverse molecular arrangements in both heterochiral and homochiral systems. Figure S4 picks up some typical tetramers with relatively high formation probabilities.

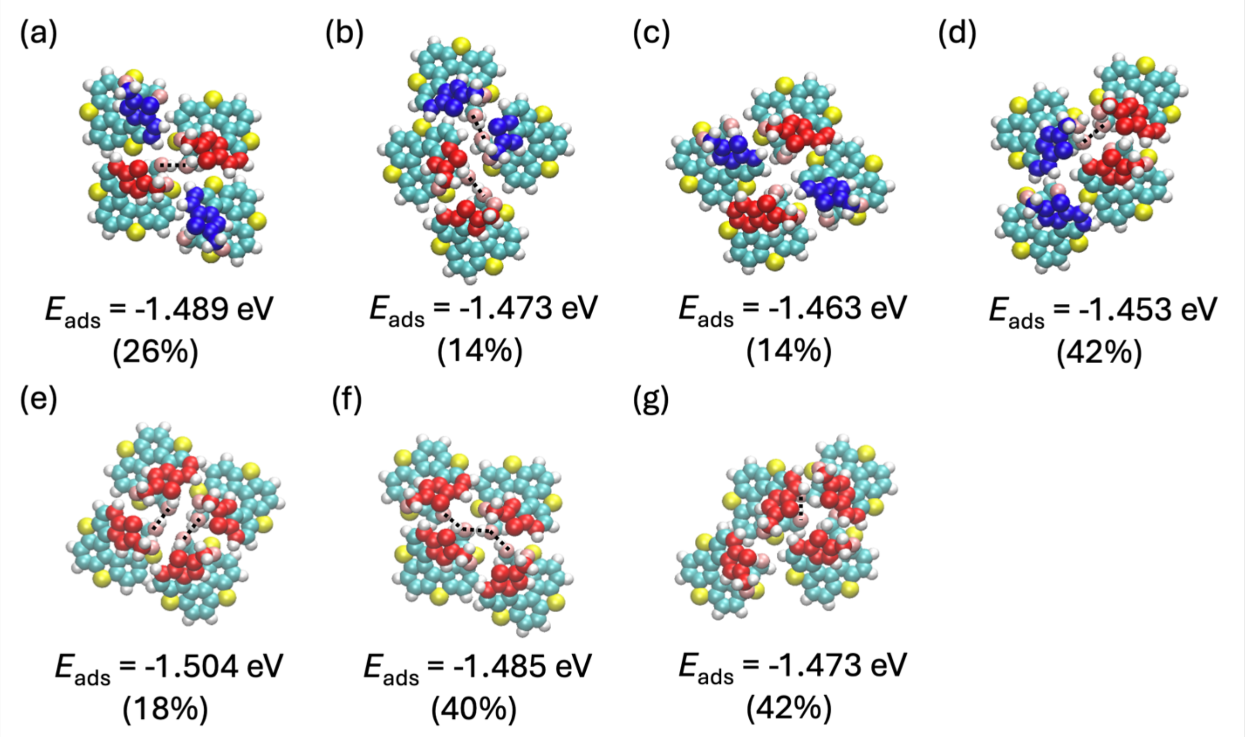

Figure S4. (**a**)–(**d**) Heterochiral and (**e**)–(**g**) homochiral tetramers of [7]TH-diol. The black dashed lines show hydrogen bonds forming between the molecules.

5. Chain structures after equilibration at 1K.

Initially, the molecules were uniformly distributed in the chain models. Here, we show the most stable chain structures after equilibration at 1 K for 500 ps: Racemic chain with intermolecular distance of 12.28 Å, and enantiopure chain with intermolecular distance of 10.44 Å. In the case of racemic chain, the molecules remained uniformly distributed, while the distance between the neighboring molecules decrease. In the case of enantiopure chain, the molecules formed hydrogen bonds between the neighboring hydroxymethyl groups. Because the graphene periodicity was not completely commensurate with chain periodicity, (M)-type molecules did not maintain uniform distribution along the chain.

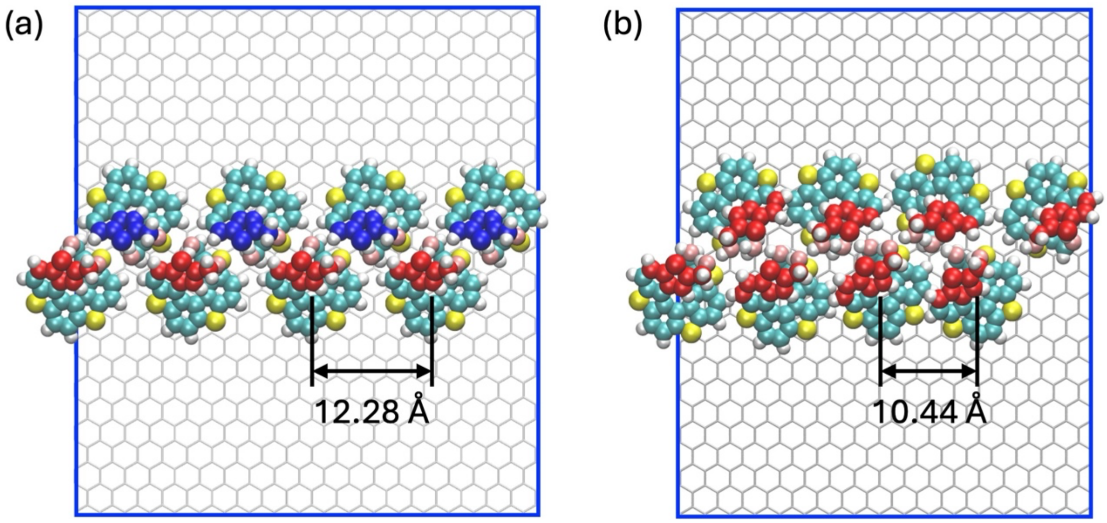

Figure S5. The optimized chain structures after equilibration at 1 K. (**a**) Racemic chain with intermolecular distance of 12.28 Å, and (**b**) enantiopure chain with intermolecular distance of 10.44 Å.

6. Partial atomic charges of [7]TH-diol.

We modified the strength of Coulomb interactions (electrostatics) by using different partial charge populations to tune the hydrogen bond strength.

The AM1-BCC method [1,2], which is widely regarded as a standard and reliable approach for parameterizing the GAFF force filed, was used as the reference for this study, because we expected it to provide the most accurate and consistent results. To systematically examine the effect of hydrogen bond strength, we also calculated alternative partial charges. The lowest-energy molecular geometries were first optimized using DFT calculation at the B3LYP/6-311++G(d,p) level with Gaussian09 software. Based on the optimized structures, atomic charges were then calculated using the Mulliken method.

Although Mulliken charges are sensitive to the choice of basis set and often underestimate partial charges for polar bonds when diffusion functionals are included [3], we employed a large basis set to obtain relatively small partial charges for atoms in the –OH groups for comparison. Both racemic and enantiopure chain models using Mulliken charges were equilibrated at 1 K, however, in this case, the chain structures were disrupted, there was no hydrogen bonds forming between the moleclues, and even molecular overlap occured between neighbors. This indecates that the chain structures are not stable when–OH groups carrying small partial charges. These results further confirm the partial charges have a significant impact on the formation and stability of ordered molecular structures.

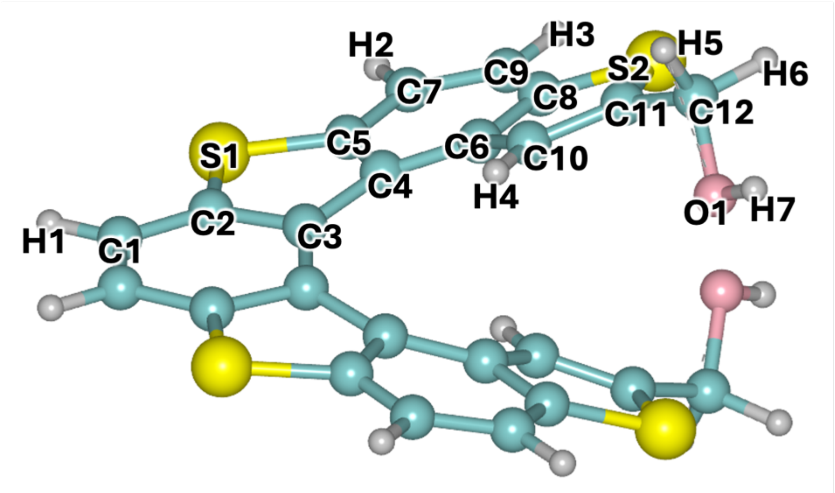

Figure S6. Ball-and-stick models of [7]TH-diol optimized using DFT at the B3LYP/6-311++G(d,p) level. Atom IDs are assigned to each atom in the molecule. Due to the C_2 symmetry of the helical structure, atoms at symmetry-equivalent positions share the same partial charges.

Table S1. Partial atomic charges of [7]TH-diol.

Atom id	AM1-BCC	Mulliken	Atom id	AM1-BCC	Mulliken
C1	-0.0890	-0.1190	S1	0.0422	-0.7929
C2	-0.0691	-0.1213	S2	0.0315	-0.1555
C3	-0.0060	0.5926	H1	0.1430	0.1799
C4	-0.0170	0.4342	H2	0.1430	0.1763
C5	-0.0631	-0.0354	H3	0.1420	0.1901
C6	-0.0388	0.4581	H4	0.1780	0.2392
C7	-0.0940	-0.1570	H5	0.0572	0.1712
C8	-0.0951	-0.0720	H6	0.0572	0.2110
C9	-0.0820	-0.0398	H7	0.4070	0.2487
C10	-0.1092	-0.4232	O1	-0.5888	-0.2352
C11	-0.1476	-0.4065			
C12	0.1986	-0.3435			

Reference

1. Jakalian, A.; Jack, D.B.; Bayly, C.I. Fast, efficient generation of high-quality atomic charges. AM1-BCC model: II. Parameterization and validation. *J. Comput. Chem.* **2002**, 23, 1623–1641. https://doi.org/10.1002/jcc.10128.
2. Jakalian, A.; Bush, B.L.; Jack, D.B.; Bayly, C.I. Fast, Efficient Generation of High-Quality Atomic Charges. AM1-BCC Model: I. Method. *J. Comput. Chem.* **2000**, 21, 132–146. https://doi.org/10.1002/(SICI)1096-987X(20000130)21:2<132::AID-JCC5>3.0.CO;2-P.
3. North SC, Jorgensen KR, Pricetolstoy J and Wilson AK (2023) Population analysis and the effects of Gaussian basis set quality and quantum mechanical approach: main group through heavy element species. *Front. Chem.* 11:1152500. https://doi.org/10.3389/fchem.2023.1152500.
